# Structural patterns of selection and diversity for *Plasmodium vivax* antigens DBP and AMA1

**DOI:** 10.1186/s12936-018-2324-3

**Published:** 2018-05-02

**Authors:** Andrew J. Guy, Vashti Irani, Jack S. Richards, Paul A. Ramsland

**Affiliations:** 1 Life Sciences, Burnet Institute, 85 Commercial Road, Melbourne, VIC 3004 Australia; 2 Department of Immunology, Monash University, Melbourne, Australia; 3 Department of Medicine, University of Melbourne, Melbourne, Australia; 4 Department of Infectious Diseases, Monash University, Melbourne, Australia; 5 Victorian Infectious Diseases Service, Royal Melbourne Hospital, Melbourne, Australia; 6 School of Science, RMIT University, Plenty Road, Bundoora, VIC 3083 Australia; 7 Department of Surgery Austin Health, University of Melbourne, Heidelberg, Australia

**Keywords:** *Plasmodium vivax*, Protein structure, Immune selection, Malaria, Population genetics

## Abstract

**Background:**

*Plasmodium vivax* is a significant contributor to the global malaria burden, and a vaccine targeting vivax malaria is urgently needed. An understanding of the targets of functional immune responses during the course of natural infection will aid in the development of a vaccine. Antibodies play a key role in this process, with responses against particular epitopes leading to immune selection pressure on these epitopes. A number of techniques exist to estimate levels of immune selection pressure on particular epitopes, with a sliding window analysis often used to determine particular regions likely to be under immune pressure. However, such analysis neglects protein three-dimensional structural information. With this in mind, a newly developed tool, BioStructMap, was applied to two key antigens from *Plasmodium vivax*: *Pv*AMA1 and *Pv*DBP Region II. This tool incorporates structural information into tests of selection pressure.

**Results:**

Sequences from a number of populations were analysed, examining spatially-derived nucleotide diversity and Tajima’s D over protein structures for *Pv*AMA1 and *Pv*DBP. Structural patterns of nucleotide diversity were similar across all populations examined, with Domain I of *Pv*AMA1 having the highest nucleotide diversity and displaying significant signatures of immune selection pressure (Tajima’s D > 0). Nucleotide diversity for *Pv*DBP was highest bordering the dimerization and DARC-binding interface, although there was less evidence of immune selection pressure on *Pv*DBP compared with *Pv*AMA1. This study supports previous work that has identified Domain I as the main target of immune-mediated selection pressure for *Pv*AMA1, and also supports studies that have identified functional epitopes within *Pv*DBP Region II.

**Conclusions:**

The BioStructMap tool was applied to leading vaccine candidates from *P. vivax*, to examine structural patterns of selection and diversity across a number of geographic populations. There were striking similarities in structural patterns of diversity across multiple populations. Furthermore, whilst regions of high diversity tended to surround conserved binding interfaces, a number of protein regions with very low diversity were also identified, and these may be useful targets for further vaccine development, given previous evidence of functional antibody responses against these regions.

**Electronic supplementary material:**

The online version of this article (10.1186/s12936-018-2324-3) contains supplementary material, which is available to authorized users.

## Background

*Plasmodium vivax* infected an estimated 8.55 million people in 2016 and is a significant contributor to global malaria morbidity, with the majority of *P. vivax* cases occurring within South-East Asia [1]. There remains a significant need for a vaccine against *P. vivax*, and an understanding of the targets of natural immune responses following *P. vivax* infection is likely to aid such an effort. A key challenge in vaccine development is the identification of specific antigens and epitopes that are targets of protective antibody responses. It is possible to use population genetic data to identify regions of proteins that are under immune-mediated selection pressure, which gives rise to balancing selection within that protein region. Tajima’s D is one test statistic that is often used to identify departures from a neutral model of selection, and has been applied to malaria genes both on a per-gene basis [2, 3], or as a sliding window analysis along a gene [4–7]. A number of studies have previously examined *P. vivax* proteins such as apical membrane antigen 1 (*Pv*AMA1) and Duffy-binding protein (*Pv*DBP) for evidence of immune selection pressure using these approaches [4, 8–11]. However, because a sliding window analysis is typically performed over the linear gene sequence, it does not take into account the impact of the three-dimensional (3D) structural constraints of the protein in the calculation of selection pressures. A new method that allows incorporation of protein structural information into tests for selection pressure has recently been described [12], and has been applied here to two leading *P. vivax* vaccine candidates: *Pv*AMA1 and *Pv*DBP.

AMA1 is a type I transmembrane protein present in all *Plasmodium* species [13]. It is localized to the parasite micronemes, and is released onto the surface of the merozoite prior to invasion of red blood cells [14]. AMA1 binds to RON2 during the formation of the tight junction between parasite and host–cell membranes [15, 16] and is a target of protective immune responses [17–20]. The ectodomain of *Plasmodium* AMA1 proteins is divided into three domains, termed Domains I (DI), II (DII) and III (DIII) (Additional file 1) [21]. DI is considered to be the most polymorphic, and is also the site of RON2 binding [22]. RON2 binds a conserved hydrophobic cleft that is surrounded by a number of highly polymorphic regions, the most notable being the C1L loop, a surface exposed loop with high variability that is suggested to define strain-specificity in anti-AMA1 responses in *Plasmodium falciparum* infection [23]. While DI is generally considered to be the most important for functional antibody responses, there is evidence that DII and DIII may also be targets of functional antibody responses in *P. falciparum* [8, 24]. A number of studies have investigated selection pressures on *Pv*AMA1. Evidence for balancing selection within Domain I has been observed in a Venezuelan population [9], in a number of Papua New Guinean populations (Madang and Wosera, Madang and East Sepik) [4, 8], an Iranian population [10] and in a Peruvian population [25]. In contrast, two other studies examining *Pv*AMA1 sequences in isolates from Korea [26] and Myanmar [27] did not find any evidence of balancing selection, but instead observed evidence of recent population bottleneck and expansion in those populations.

*Pv*DBP is an important micronemal protein that binds to the Duffy antigen/receptor for chemokines (DARC) on human reticulocytes during invasion [28, 29]. Whilst there is evidence that *Pv*DBP is not absolutely essential for invasion of reticulocytes [30–32], Duffy-negative individuals are largely resistant to *P. vivax* infection, and hence *Pv*DBP makes an attractive vaccine target [33]. *Pv*DBP is part of the erythrocyte-binding like (EBL) family of proteins, which include EBA175, EBA181 and EBA140 in *P. falciparum* [34]. *Pv*DBP is the sole EBL family protein in *P. vivax* [35]. EBL family proteins are composed of a number of distinct domains, with Region II (RII) being a cysteine-rich Duffy-binding like (DBL) domain that is involved in binding to erythrocytes. EBL family proteins each recognize a different receptor via their respective DBL domains [29, 36–38]; *Pv*DBP binds to DARC via its DBL domain (RII) [39]. During this process two *Pv*DBP molecules form a dimer around two DARC molecules [39, 40]. RII of *Pv*DBP has been divided into a number of subdomains (subdomains 1–3) [41] (Additional file 2), and it is subdomain 2 that contains both the dimer interface and DARC binding residues [39]. Immune responses against *Pv*DBP have been associated with protection from clinical malaria in naturally exposed cohorts [42, 43] whilst antibodies against *Pv*DBP RII epitopes have been found to inhibit both attachment of *Pv*DBP RII to erythrocytes [44] and in vitro invasion of erythrocytes [45]. With regards to immune selection pressure on *Pv*DBP, a study of 100 Sri Lankan isolates found no evidence of significant selection pressure on this region using Tajima’s D, dN/dS or Fu and Li’s D and F statistics [11]. Another study examining genetic diversity of *Pv*DBP RII across multiple populations showed a significantly positive value of dN/dS in this region, suggesting that this region may be under immune selection pressure [25].

In this study, selection pressures on *Pv*AMA1 and *Pv*DBP Region II were examined in the context of protein structure, using a newly developed tool called BioStructMap [12]. BioStructMap enables the application of a 3D sliding window over a protein structure. This allows incorporation of protein structural information into tests such as Tajima’s D or nucleotide diversity that are traditionally performed as a linear 2D sliding window over a protein or nucleotide sequence. A previous study identified a discontinuous region of *Pf*AMA1 bordering DII and DIII that had a strong signature of balancing selection when considering spatially derived Tajima’s D [12]. Given that other studies have identified DI of *Pv*AMA1, rather than DII or DIII, as being under balancing selection, it was considered that incorporation of protein structural information might yield additional insights into other regions under immune selection pressure. Genomic sequences from a number of populations were analysed, and spatially-derived nucleotide diversity and Tajima’s D were examined using protein structural information for *Pv*AMA1 and *Pv*DBP Region II. Structural patterns of nucleotide diversity were similar across all populations examined, with Domain I of *Pv*AMA1 having the highest nucleotide diversity and displaying significant signatures of balancing selection (Tajima’s D > 0). Nucleotide diversity for *Pv*DBP was highest bordering the dimerization and DARC-binding interface, although there was less evidence of immune selection pressure on this antigen.

## Methods

### Data sources

Reference sequences for Sal-1 *Pv*DBP (PVX_110810) and *Pv*AMA1 (PVX_092275) were obtained from PlasmoDB, v34 (http://www.plasmoDB.org) [46]. Genomic sequences from field isolates were extracted from GenBank, restricted to *P. vivax*, and with the condition that sequences had to cover > 95% of the structured domains examined here (i.e. partial fragments from these regions were excluded). Sequences from non-human hosts were excluded, as were sequences without a known geographic location described either in the associated literature or clearly annotated in the sequence record. Single isolate populations were also excluded. For *Pv*DBP, bases 766–1659 were used, while for *Pv*AMA1 bases 121–1422 were used.

### Sequence polymorphism analysis

The DendroPy Python library [47] was used to calculate Tajima’s D, Watterson’s Theta, mean pairwise differences and nucleotide diversity. Haplotype diversity was calculated using:

$$ H = \frac{n}{n - 1}\left( {1 - \mathop \sum \limits_{i} x_{i}^{2} } \right) $$

where *x*_i_ is the relative frequency of the *i*th haplotype and *n* is the number of samples.

Normalized Shannon entropy [48] for each position in the protein sequence was calculated using:

$$ S = - \;\mathop \sum \limits_{i} \frac{{p_{i} \;log_{2} \;p_{i} }}{{log_{2} \;20}} $$

where S is the normalized Shannon entropy and *p*_*i*_ is the frequency at that position of the *i*th amino acid in the standard 20 amino acid alphabet. The normalized Shannon entropy is a measure of sequence diversity, and takes values between 0 and 1, where 0 indicates perfect sequence conservation at that site, and 1 indicates an even distribution of all possible amino acids at that site.

### Phylogenetic analysis

Sequences from all isolates were aligned using MUSCLE v3.8.31 [49], and alignments manually adjusted to minimize gaps. Maximum likelihood phylogenetic trees were constructed for AMA-1 and DBP sequences using IQ-TREE v1.3.11.1 [50]. The ultrafast bootstrap estimation (UFBoot) [51] was used with 5000 bootstrap replicates and a best fit model was chosen according to the Bayesian Inference Criterion [52]. Phylogenetic trees were visualized with iTOL [53].

### Incorporation of structural information into sequence analysis

A Python package, BioStructMap, which allows for the application of a 3D sliding window over a protein structure has been previously described [12]. BioStructMap was used to compute spatially derived Tajima’s D and nucleotide diversity (π) values for both PvAMA1 and PvDBP structures, with a radius of 15 Å for each window. BioStructMap was also used to calculate Normalized Shannon Entropy on a per-residue basis.

### Protein structural models

Known *Pv*DBP structures (i.e. 4NUU, 3RRC, 4YFS, 4NUV, 5F3J) all contain a number of unresolved residues, and to ensure complete coverage of DBP Region II in this analysis, a template-based model of *P. vivax* DBP was generated for use with BioStructMap. This model was created using ModPipe [54], an automated software pipeline that utilizes MODELLER for the generation of comparative protein structure models [55]. The PDB structure 4NUU was used to generate this comparative model. The generated model is accessible via ModBase (https://modbase.compbio.ucsf.edu/; ModPipe model ID f7602e019fac5be4a79c4cca6751b392) [56].

The *Pv*AMA1 model used has been previously described [4], and uses a chimeric template to generate a structural model of *Pv*AMA1.

### Comparing patterns of selection and diversity between populations

To compare structural patterns of nucleotide diversity and Tajima’s D between all populations considered in this study, Spearman’s rank correlation coefficient was computed for both *Pv*AMA1 and *Pv*DBP residue data between each pair of populations. Residues with missing data in one or both populations (i.e. Tajima’s D was undefined) were excluded from analysis for that pair of populations.

### Data analysis, statistics and other software used

The majority of data analysis was performed using the Anaconda distribution of Python 3.5. Plotting was performed with the Python Matplotlib package, version 1.5.1 [57]. Statistical analysis was performed using SciPy [58]. Protein structures were visualized using PyMol [59].

## Results

### Population structure of PvAMA1 and PvDBP sequences

**Fig. 1:**
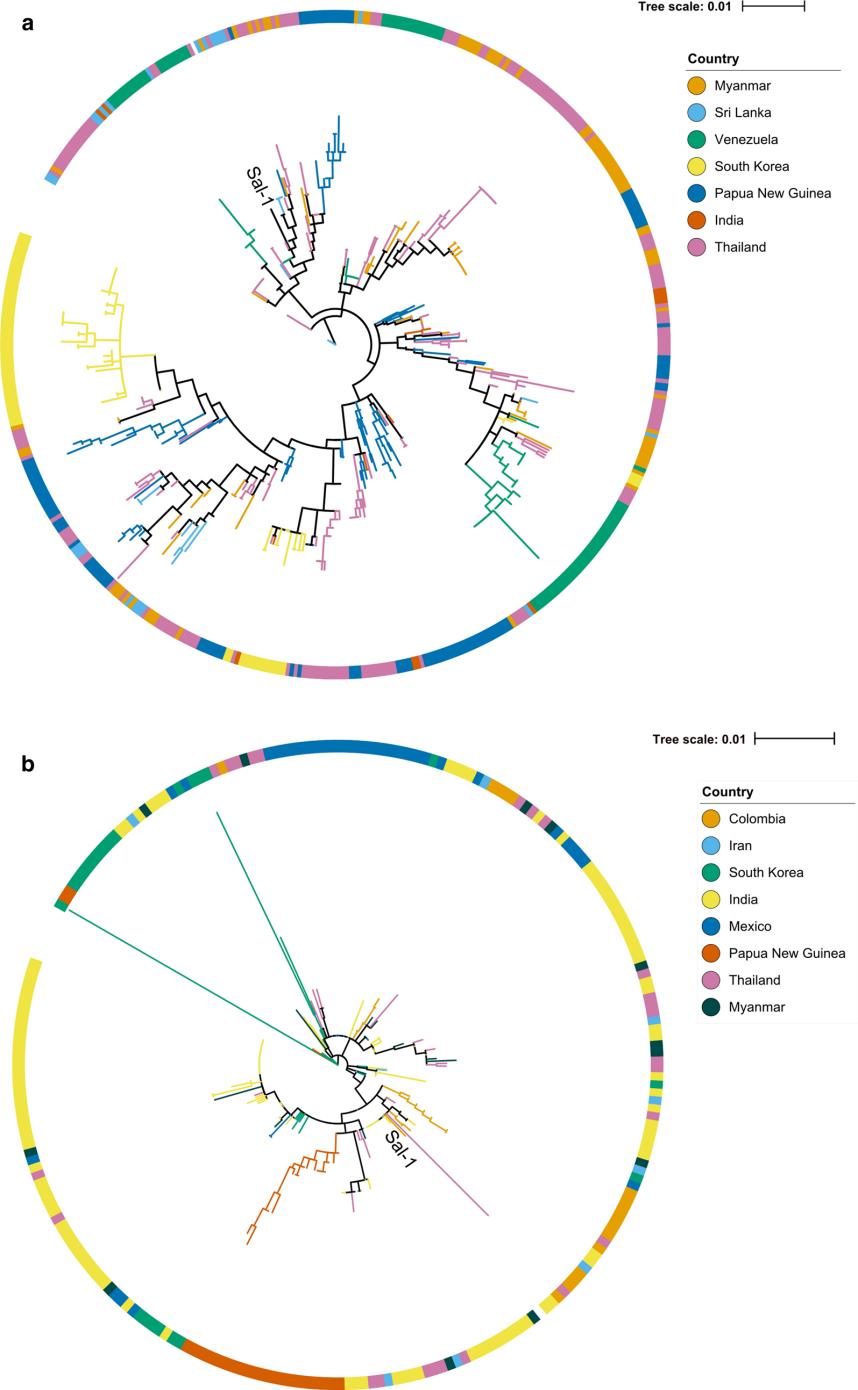
Population structure of *Pv*AMA1 and *Pv*DBP RII sequences. Maximum-likelihood phylogenetic trees are shown for *Pv*AMA1 (**a**) and *Pv*DBP Region II (**b**). Leaves are coloured according to the geographic location for each strain. The location of the Sal-1 reference strain is also indicated on each figure

This study aimed to examine selection pressures on key structured domains of *Pv*DBP and *Pv*AMA1. Genomic sequences for each antigen were extracted from GenBank, with a total of 505 *Pv*AMA1 sequences and 243 *Pv*DBP sequences obtained, belonging to 10 and 12 distinct populations, respectively. Sequences which did not cover at least 95% of the structured domains examined (*Pv*AMA1, PVX_092275: nucleotides 121–1422; *Pv*DBP, PVX_110810: nucleotides 766–1659) were excluded from analysis, as were sequences from single-isolate populations or non-human hosts. Maximum likelihood phylogenetic trees were constructed for both *Pv*AMA1 and *Pv*DBP (Fig. 1) using aligned sequences from all populations. Some populations were generally contained on their own branch (e.g. South Korea for *Pv*AMA1, Mexico and Papua New Guinea for *Pv*DBP), while other branches contained a mix of populations. This intermixing was particularly evident for populations that are geographically close, such as Thailand, Myanmar and Papua New Guinea for *Pv*AMA1.

### Traditional measures of selection pressure and diversity for PvAMA1 and PvDBP

Key population parameters for *Pv*AMA1 and *Pv*DBP sequences were computed, as well as several measures of diversity and selection (Tables 1, 2). A total of 259 haplotypes were observed for *Pv*AMA1 (haplotype diversity = 0.99), while 84 haplotypes were observed for *Pv*DBP RII (haplotype diversity = 0.96).

**Table 1:** Population genetics parameters for PvAMA1 sequences from various geographic locations

Country	n	Number of polymorphic sites	NS sites	S sites	π (× 10^−3^)	k	θ	Tajima’s D	H	Hd
Myanmar	73	43	31	12	8.55	10.90	8.85	0.75	36	0.935
Sri Lanka	23	34	29	5	7.78	10.13	9.21	0.38	15	0.949
Papua New Guinea (Madang)	61	36	33	4	8.76	11.41	7.69	1.59	51	0.992
Papua New Guinea (East Sepik)	41	34	27	7	8.02	10.45	7.95	1.09	37	0.995
Thailand (Tak; 1996)	58	48	36	12	9.69	12.62	10.37	0.73	52	0.995
Thailand (Tak; 2007)	44	46	33	13	10.09	13.13	10.57	0.85	31	0.982
Thailand (Chantaburi)	56	44	34	10	10.02	13.04	9.58	1.22	25	0.895
India	10	27	22	5	8.31	10.82	9.54	0.64	7	0.911
South Korea	66	57	36	21	5.90	7.68	11.98	− 1.20	17	0.921
Venezuela	73	29	22	7	7.66	9.98	5.97	2.12*	17	0.908

*n* number of isolates, *NS sites* number of sites with non-synonymous nucleotide polymorphisms, *S sites* number of sites with synonymous nucleotide polymorphisms, *π* nucleotide diversity, *k* mean number of pairwise differences, *θ* Watterson’s theta, *H* number of haplotypes, *Hd* haplotype diversity

* p < 0.05, indicating rejection of the null hypothesis of a neutral mutation model (confidence limits from Tajima, 1989)

**Table 2:** Population genetics parameters for PvDBP sequences from various geographic locations

Country	n	Number of polymorphic sites	NS sites	S sites	π (× 10^−3^)	k	θ	Tajima’s D	H	Hd
Papua New Guinea	23	18	14	5	5.24	4.69	4.88	− 0.14	11	0.925
Thailand (Bangkok)	25	46	39	9	10.37	9.01	12.18	− 1.00	19	0.977
Colombia	17	14	12	2	6.71	6.00	4.14	1.72	16	0.993
Myanmar	12	32	25	7	10.17	9.09	10.60	− 0.64	10	0.970
Mexico	35	13	9	4	3.38	3.02	3.16	− 0.14	8	0.556
India (Panna)	20	22	20	2	5.89	5.23	6.20	− 0.60	9	0.858
India (Chennai)	20	18	16	2	6.60	5.86	5.07	0.58	8	0.842
India (Delhi)	20	19	16	3	6.64	5.90	5.36	0.38	11	0.889
India (Nadiad)	20	21	17	4	6.54	5.81	5.92	− 0.07	10	0.921
India (Kamrup)	20	23	20	3	7.81	6.94	6.48	0.27	12	0.942
Iran	8	21	18	3	8.81	7.57	8.10	− 0.34	8	1.000
South Korea	23	74	58	17	10.53	9.36	20.05	− 2.12*	11	0.854

*n* number of isolates, *NS sites* number of sites with non-synonymous nucleotide polymorphisms, *S sites* number of sites with synonymous nucleotide polymorphisms, *π* nucleotide diversity, *k* mean number of pairwise differences, *θ* Watterson’s theta, *H* number of haplotypes, *Hd* haplotype diversity

* p < 0.05, indicating rejection of the null hypothesis of a neutral mutation model (confidence limits from Tajima, 1989)

For *Pv*AMA1, nucleotide diversity (π) and mean number of pairwise differences (k) were highest in the three Thai populations examined, with a maximum π of 10.09 × 10^−3^ in the 2007 Tak Province, Thailand samples. Nucleotide diversity was lowest in the South Korean population (π = 5.90 × 10^−3^), which may be explained by a recent bottleneck in this population, limiting overall diversity; this is supported by the negative Tajima’s D value observed for *Pv*AMA1 in this South Korean population (D = − 1.20). Tajima’s D is often used to identify regions under balancing selection (Tajima’s D > 0), which can be the result of immune selection pressure, and it is noted that for *Pv*AMA1, only the Venezuelan population had a significantly positive Tajima’s D value (D = 2.12, p < 0.05), as per the confidence limits outlined in Tajima [60], although these limits may be overly conservative [61]. Most other populations also had a positive Tajima’s D value, such as Papua New Guinea (Madang: D = 1.59; East Sepik: D = 1.09) and Thailand (Chantaburi: D = 1.22), with the sole exception of South Korea (discussed above).

When examining *Pv*DBP, nucleotide diversity was highest in South Korea (π = 10.53 × 10^−3^), Bangkok, Thailand (π = 10.37 × 10^−3^) and Myanmar (π = 10.17 × 10^−3^). Interestingly, the South Korean population had a negative Tajima’s D value that was statistically significant, suggestive of a recent population bottleneck and expansion. This population also had the highest number of polymorphic sites (58 non-synonymous). This high variability is probably due to 2 or 3 divergent isolates (Fig. 1b), and haplotype diversity is relatively low for this population (Hd = 0.85). Tajima’s D values for *Pv*DBP were mostly close to zero, with the highest value observed for a Colombian population (D = 1.72).

These observations agree with previous studies that have generally observed greater signatures of immune selection pressure on *Pv*AMA1 as compared to *Pv*DBP [25].

### Diversity and selection on the PvAMA1 structure

**Fig. 2:**
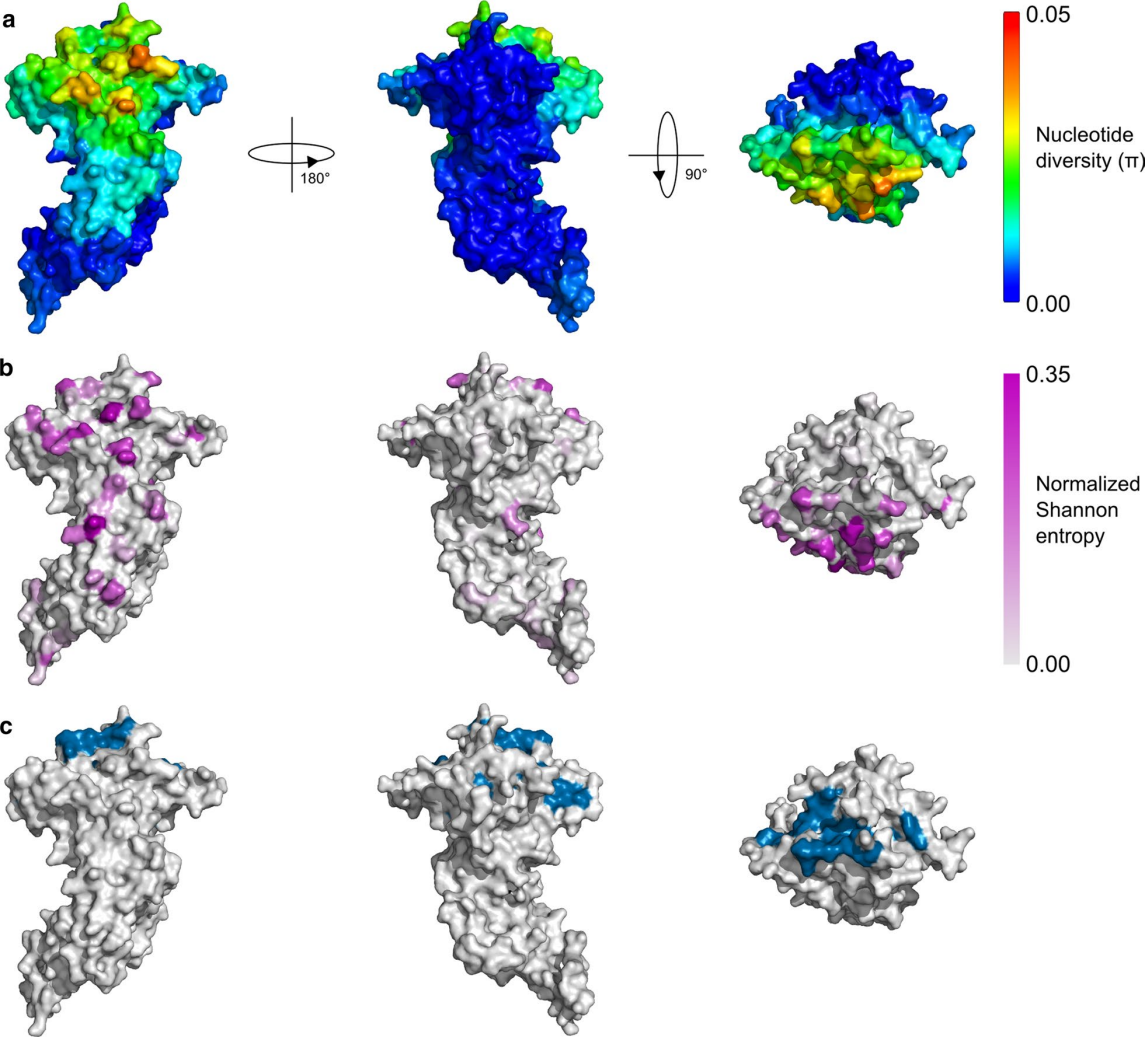
Measures of sequence diversity for *Pv*AMA1 and relationship to key binding interfaces. **a** Spatially-derived nucleotide diversity (π) for *Pv*AMA1 displayed over the modelled *Pv*AMA1 structure. A radius of 15 Å was used for each 3D sliding window. **b** Normalized Shannon entropy for *Pv*AMA1 residues on a per-site basis, with no spatial averaging performed. Higher entropy values are indicative of greater sequence diversity across all strains at that residue position. **c** Residues involved in the binding of *Pv*AMA1 to its *Pv*RON2 ligand. Residues within 4 Å of the RON2 peptide are shown in blue on the modelled *Pv*AMA1 structure (as defined by the PDB structure 5NQG). Note that some binding residues are not visible due to the presence of the flexible Domain II loop near the RON2 binding groove

We then examined selection pressures and polymorphisms in the context of protein 3D structure using BioStructMap, a tool that allows for incorporation of protein structural information into a sliding window analysis. To quantify nucleotide diversity over the *Pv*AMA1 structure both as a spatially averaged value and a per-residue value, nucleotide diversity was calculated using a 3D sliding window (Fig. 2a) and per-residue normalized Shannon entropy (Fig. 2b) using sequences from all populations. Normalized Shannon entropy is a measure of sequence diversity, taking values between 0 and 1, where 0 indicated perfect sequence conservation at that position, while 1 indicates an even distribution of all possible amino acids at that position. For *Pv*AMA1, nucleotide diversity was highest in DI on one side of the RON2 binding cleft (Fig. 2a, c). There was limited diversity within DII, and very little observed in DIII. This pattern of nucleotide diversity appeared to be maintained when examining patterns of nucleotide diversity in individual populations (Additional file 3), with the exception of the South Korean population, in which even DI displays limited diversity. This is possibly due to recent bottleneck and expansion, as suggested by the negative Tajima’s D value for this population. It was also observed that the so-called ‘silent face’ of *Pv*AMA1 had very low diversity in all populations examined, which is in line with a number of other studies that have noted a distinct lack of polymorphisms on this face both in *P. falciparum* [8, 62–64] and *P. vivax* [4].

**Fig. 3:**
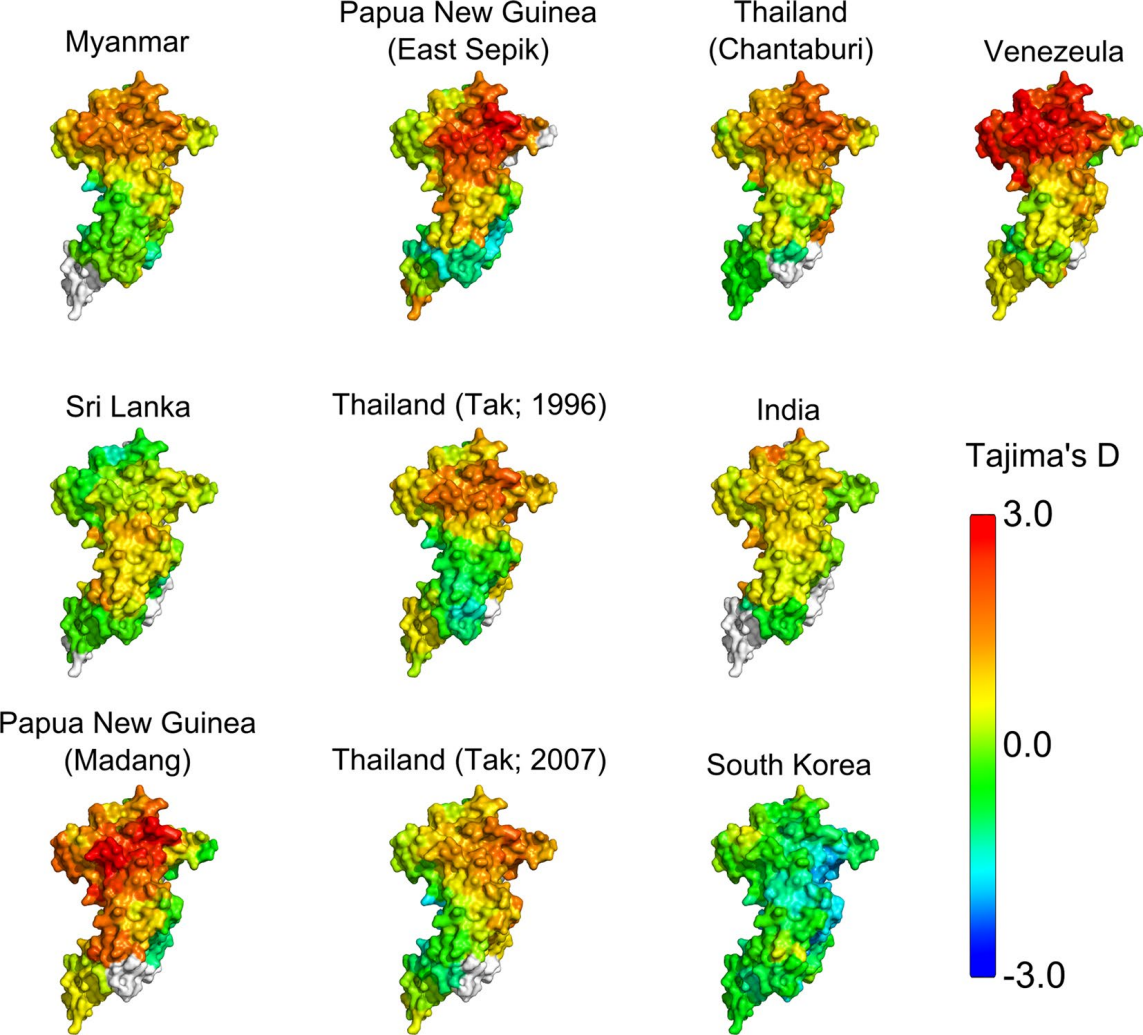
Spatially derived Tajima’s D plotted for *Pv*AMA1 across various populations. Tajima’s D was calculated using a 3D sliding window over a modelled *Pv*AMA1 structure, with a radius of 15 Å for each window

To test for evidence of immune-mediated selection pressure on *Pv*AMA1, Tajima’s D was calculated as a 3D sliding window over the modelled protein structure (referred to as a spatially-derived Tajima’s D). A positive Tajima’s D value provides evidence for balancing selection, which can arise as a result of immune pressure. It is noted that departures from neutrality can also arise as a result of population structure. In particular, sampling of strains across multiple distinct populations can potentially give rise to similar signatures to those of balancing selection. For this reason Tajima’s D values were analysed within distinct geographic and temporal populations. Spatially-derived Tajima’s D was highest in DI for nearly all populations examined, with the exception of Sri Lanka and South Korea (Fig. 3; Additional file 4). As was observed for nucleotide diversity, Tajima’s D was highest on one side of DI, with the other ‘silent-face’ typically having Tajima’s D values close to zero. This is in agreement with several studies, both in *P. vivax* and *P. falciparum*, in which polymorphisms on AMA1 are focused on one side of the protein structure, with minimal polymorphic variation on the other face [4, 8, 62–64]. It has been hypothesized that this silent face of the protein is not exposed to the immune system during the invasion process due to interaction with other parasite proteins, or is otherwise inaccessible to antibody binding [63].

### Diversity and selection on the PvDBP structure

**Fig. 4:**
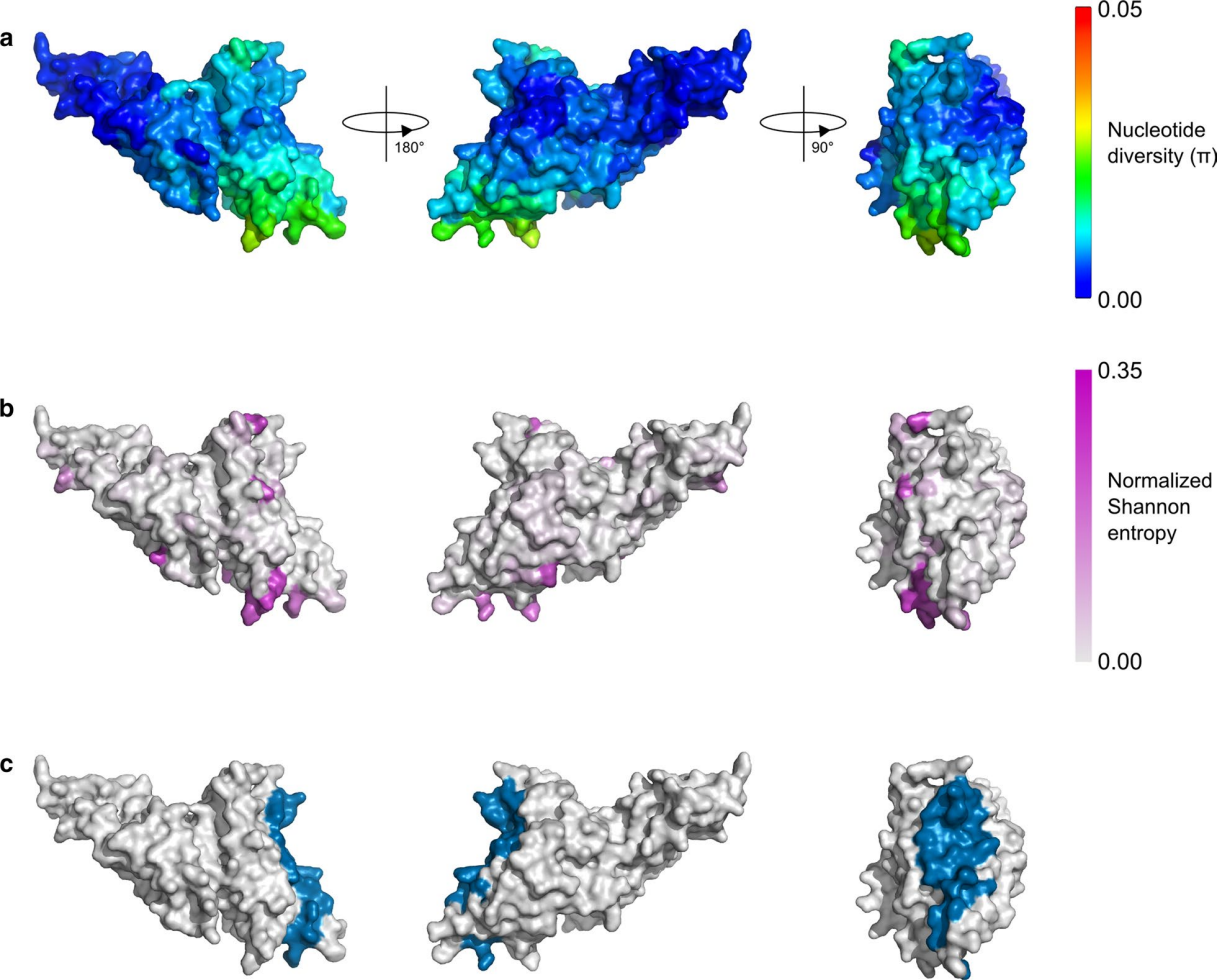
Measures of sequence diversity for *Pv*DBP and relationship to key binding interfaces. **a** Spatially-derived nucleotide diversity (π) for *Pv*DBP displayed over the modelled *Pv*DBP structure. A radius of 15 Å was used for each 3D sliding window. **b** Normalized Shannon entropy for *Pv*DBP residues on a per-site basis, with no spatial averaging performed. Higher entropy values are indicative of greater sequence diversity across all strains at that residue position. **c** Residues involved in the *Pv*DBP dimerization and DARC binding interface. Residues within 4 Å of the corresponding dimer chain or the DARC ligand (as defined by the PDB structure 4NUU) are shown in blue on the modelled *Pv*DBP structure

**Fig. 5:**
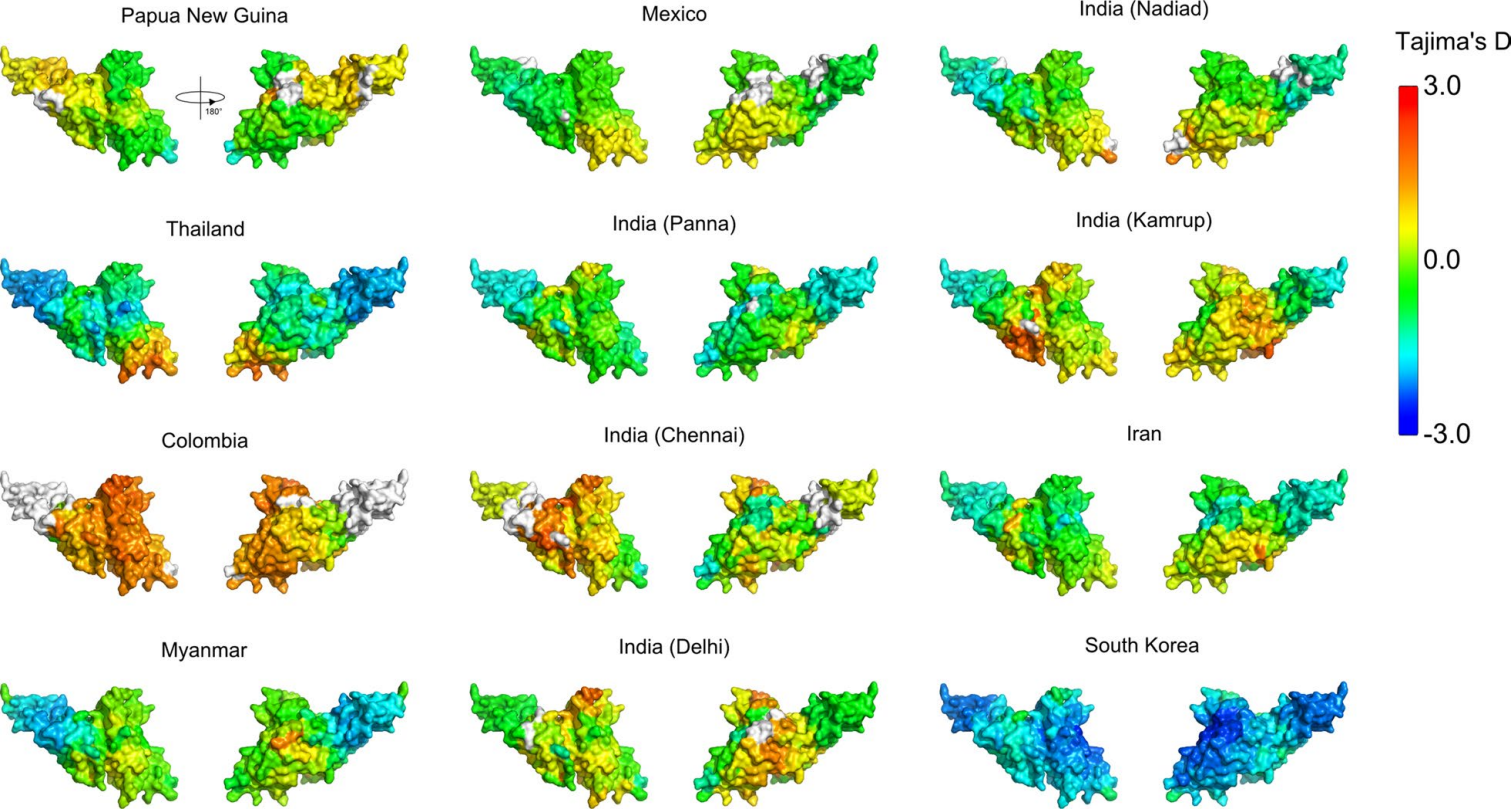
Spatially derived Tajima’s D plotted for *Pv*DBP across various populations. Tajima’s D was calculated using a 3D sliding window over a modelled *Pv*DBP structure, with a radius of 15 Å for each window

A similar analysis was performed for *Pv*DBP, calculating spatially-derived nucleotide diversity, normalized Shannon entropy and spatially-derived Tajima’s D over the modelled *Pv*DBP structure. Global nucleotide diversity was highest within subdomain 2 of *Pv*DBP, with the most polymorphic residues clustering around the dimerization and DARC binding interface (Fig. 4). Residues that are directly involved in DARC binding and dimerization were nearly all highly conserved. When examining patterns of nucleotide diversity across individual geographic locations (Additional file 5), it is noted that nucleotide diversity within *Pv*DBP RII was universally highest in a region that corresponds to a previously identified inhibitory epitope, termed the DEK epitope [33, 39, 44], which corresponds to residues 338–353 (DEKAQQRRKQWWNESK) of the Sal-1 reference sequence. Correspondingly, the opposite end of the *Pv*DBP protein (subdomain 3) had very low nucleotide diversity across all populations. Spatially derived Tajima’s D values were also calculated for *Pv*DBP, and observed that most populations had Tajima’s D values that were negative or close to zero over most of the structure (Fig. 5; Additional file 6); Tajima’s D values did not reach statistical significance in any population (with the exception of a single residue in samples from Chennai, India) (Additional file 7). However, it was noted that spatially derived Tajima’s D values were generally highest in subdomain 2.

### Amino acid mutations within binding interfaces

While binding interfaces were generally observed to have low nucleotide diversity, there were some polymorphic residues located within these binding interfaces for both *Pv*AMA1 and *Pv*DBP (Additional file 8). There were a total of 9 polymorphic residues within the *Pv*AMA1:RON2 interface, although most of these were present at very low frequencies within the global population. The three most frequent polymorphisms within the *Pv*AMA1:RON2 interface fall within a short stretch from residues 130–133 (N132D, N130K, D133N). The most frequent of these polymorphisms (N132D) is a relatively conservative change from asparagine to aspartic acid, and is the only one of the three residues whose side chains are directly involved in hydrogen bonds with RON2; the side chains from both N130 and D133 are only involved in intramolecular hydrogen bonds. It has previously been shown that a *Pv*RON2 peptide binds well to *Pf*AMA1 [22]. With this in mind, it is noted that the region corresponding to N130–D133 in *Pv*AMA1 is not conserved in *Pf*AMA1, and that this region is also not involved in the binding between *Pv*RON2 and *Pf*AMA1 (PDB structure 5NQF) [22]. These observations, coupled with the observation that a number of polymorphisms fall within this region, suggests that these residues are not a major determinant for binding to *Pv*RON2 and are hence amenable to polymorphic variation.

There were 7 polymorphic residues observed within the *Pv*DBP dimerization and DARC binding interface, although again most of these were present at low frequencies. The highest frequency polymorphism within this region is R263S, with a minor allele frequency of 20%. In the PDB structure 4NUU, the backbone of R263 is involved in hydrogen bonds to DARC, while the side chain is involved in a single intra-molecular hydrogen bond. This residue is also part of a loop which is disordered when not bound to DARC (loop 254–267) [39], suggesting a level of structural plasticity. The other polymorphic residue of note is T359R, which was identified as a major contact within the DARC binding interface by Batchelor et al. [39], although this polymorphism was observed at a relatively low minor allele frequency of 4.1% across all populations. However, this residue is not conserved between *Pv*DBP and *Pk*DBPα; *Pk*DBPα has an arginine at this position and is also able to bind DARC, supporting the viability of the T359R mutation with regards to maintaining DARC binding ability.

### Comparison between 3D and linear sliding window approaches

While a number of previous studies have utilized a linear sliding window approach when calculating Tajima’s D or nucleotide diversity [4–7, 65], this study is one of the first to utilize a spatially derived Tajima’s D. As such, the Tajima’s D values obtained using a 3D sliding window were compared with those obtained using a conventional linear sliding window, using a window size of 102 base pairs and a step size of 3 base pairs for the linear sliding window (Additional files 9, 10). In general there was broad correspondence between the two approaches for both *Pv*DBP RII and *Pv*AMA1, although there were some additional regions within *Pv*AMA1 with Tajima’s D values above the threshold for significance when using a 3D sliding window approach that were not identified with a linear sliding window. However, these regions were generally structurally contiguous with other stretches of sequence that were above the threshold for significance when using a linear sliding window.

### Comparison of spatially derived nucleotide diversity and Tajima’s D between populations

**Fig. 6:**
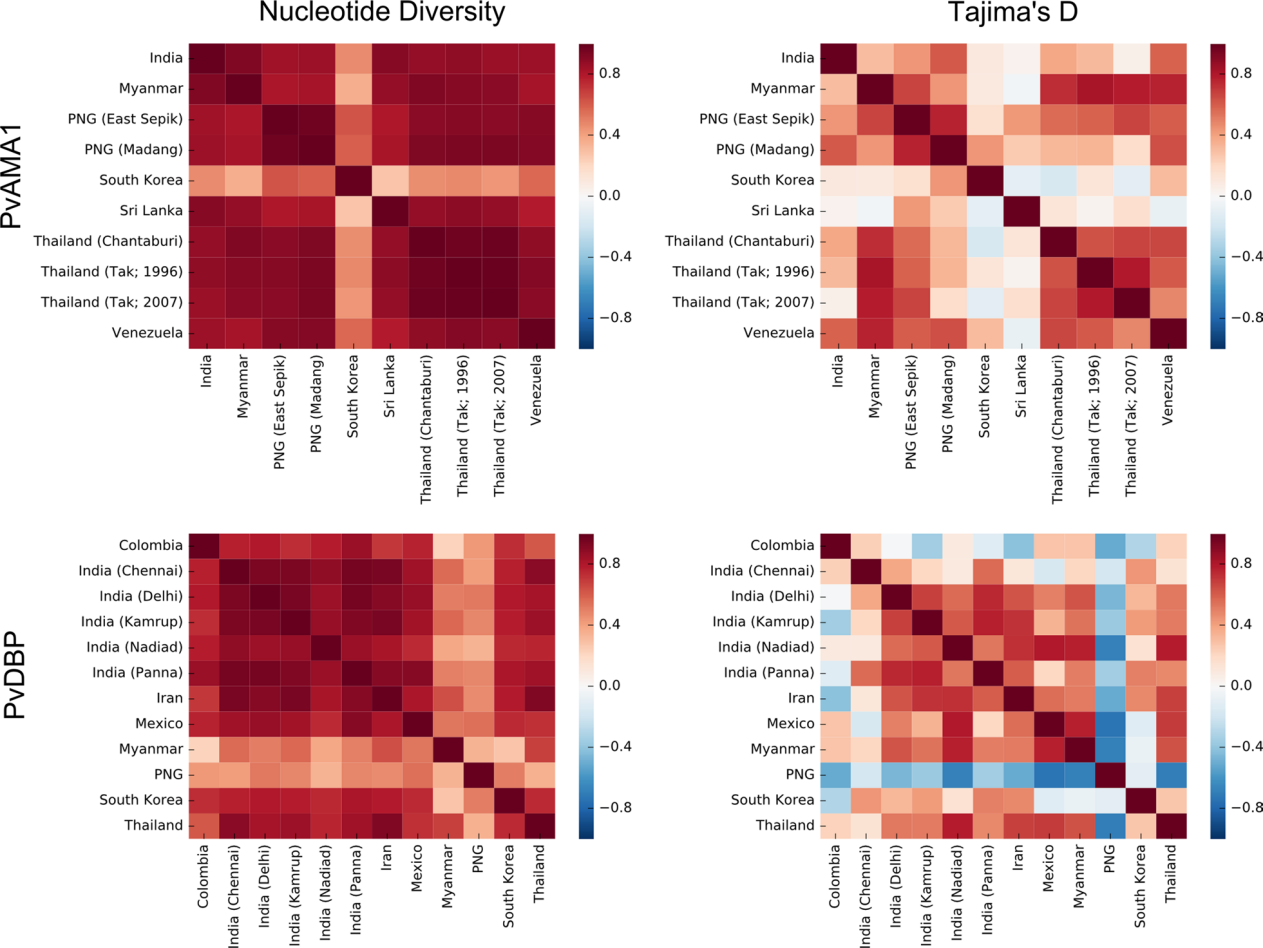
Similarity in structural patterns of nucleotide diversity and Tajima’s D between populations. Spearman’s rank correlation coefficient was calculated for each pair of populations, comparing spatially derived nucleotide diversity and Tajima’s D values between each residue in the respective protein structures

When examining differences in the patterns of nucleotide diversity and Tajima’s D values between various populations worldwide, it appeared that most populations had similar structural patterns of diversity/selection, with a few exceptions that could be due to other population effects such as recent bottleneck and expansion events. To quantify the degree of similarity between structural patterns of selection, we computed Spearman’s rank correlation coefficient for nucleotide diversity and Tajima’s D values between every pair of populations (Fig. 6). Structural patterns of nucleotide diversity were generally highly correlated between populations, with the exception of South Korea for *Pv*AMA1, and Myanmar and Papua New Guinea for *Pv*DBP. In contrast, there was less agreement in the structural patterns of Tajima’s D over each structure. There was reasonable positive correlation between most populations for spatially derived Tajima’s D over the *Pv*AMA1 structure, with the exception of South Korean and Sri Lankan populations. For *Pv*DBP, the Papua New Guinean population was the major outlier when considering spatially derived Tajima’s D values, with the highest values observed on subdomain 3, furthest from the dimerization interface. The lower level of apparent immune selection pressure on *Pv*DBP may explain the more discordant results observed between populations for *Pv*DBP; nearly all Tajima’s D values observed for *Pv*DBP do not meet the threshold for statistical significance (p < 0.05) as defined by Tajima [60] and, therefore, tests of correlation are more sensitive to small variations in Tajima’s D. In contrast, a number of populations had significantly positive spatially derived Tajima’s D values for *Pv*AMA1, including both Papua New Guinean populations (Madang, East Sepik) and the Venezuelan population (Additional file 7).

## Discussion

In this study, patterns of nucleotide diversity and selection were examined over the protein structures for the *P. vivax* antigens *Pv*AMA1 and *Pv*DBP. A number of major observations stand out from this work. Firstly, patterns of diversity on both *Pv*AMA1 and *Pv*DBP were remarkably similar across multiple geographic populations, despite phylogenetic trees for both *Pv*AMA1 and *Pv*DBP sequences suggesting a level of clustering according to geographic location. The only exception to this for *Pv*AMA1 was the South Korean population which displayed evidence of a recent bottleneck and expansion. This similarity in patterns of diversity is important when trying to extend conclusions made from studies from single geographic locations to a worldwide population, and these observations suggest a universality with regards to major epitopes on these antigens. It is also interesting to note that highly polymorphic residues for both *Pv*AMA1 and *Pv*DBP tended to fall within regions surrounding, but not a part of, ligand binding interfaces. For AMA1, RON2 binds in a hydrophobic cleft in DI, and polymorphic residues fall on one side of this hydrophobic cleft, but generally not within residues that make contact with the RON2 peptide. Similarly with *Pv*DBP, contact with DARC occurs primarily via subdomain 2, and the most polymorphic regions were near the DARC binding and dimerization interface. In contrast, the residues directly involved in the *Pv*DBP binding interface were highly conserved. These results are suggestive of two things. Firstly, the residues that make up the key binding interfaces in these two antigens have limited capacity for polymorphic variation due to functional constraints, as has been previously suggested by other studies [39, 63]. This makes them attractive vaccine targets, as potential epitopes within these binding sites would have very limited antigenic diversity, and are also less likely to undergo extensive mutations to evade immune responses. Secondly, the high degree of polymorphism around these interfaces suggests that antibody responses that target these polymorphic sites are capable of inhibiting parasite invasion. This inhibition is likely the result of steric hindrance preventing receptor binding and/or dimerization. Future efforts could involve epitope focusing techniques [66, 67] to direct antibody responses to these key conserved interfaces.

For *Pv*AMA1, we observed balancing selection primarily on DI in all populations examined. Additionally, while the 3D sliding window approach highlighted additional residues under balancing selection as compared to a linear sliding window approach, nearly all of these regions fell within DI. This agrees with a number of other studies in which *Pv*AMA DI is the only domain found to be under significant balancing selection [4, 8–10]. This is in contrast to selection pressures observed on *Pf*AMA1, in which both DI and DIII have been observed to be under balancing selection [5–9, 68]. Previous work of ours has applied spatially-derived Tajima’s D calculations over a *Pf*AMA1 structure and identified strong balancing selection in a region bordering DII and DIII [12], lending further evidence to DIII being under immune selection pressure in *P. falciparum* but not *P. vivax* AMA1. The biological reasons for such a difference are unclear, as AMA1 has a conserved role between *Plasmodium* species, although it is possible that DIII of *Pv*AMA1 is less immunogenic than the corresponding *Pf*AMA1 domain due to structural or sequence differences between the two antigens.

Although individuals in malaria endemic areas develop antibodies to *Pv*AMA1 [69–71], there are no comprehensive studies on how these antibodies interact with the different domains of *Pv*AMA1. However, AMA1 is functionally conserved across *Plasmodium* species, and there is evidence that *Pv*AMA1 is functionally equivalent in a *P. falciparum* transgenic line in which *Pf*AMA1 is replaced by *Pv*AMA1 [72]. As such, comparisons can be drawn with antibody studies on *Pf*AMA1. Dutta et al. [24] generated a panel of monoclonal antibodies (mAbs) to *Pf*AMA1 and observed that their strain specificity and functional activity was determined by the diversity of the epitope sequence. The limited diversity in *Pv*AMA1 DIII observed in this current study aligns with the observations that mAbs to *Pf*AMA1 DIII were the most strain transcending. Similarly, we observed that the polymorphic face of *Pv*AMA1 DI had the highest diversity, in line with the observation by Dutta et al. that mAbs that bound the polymorphic face of *Pf*AMA1 DI were strain specific, compared to the others that bound the conserved face [24]. Importantly, mAbs that bound to the conserved face of *Pf*AMA1 still showed strong growth inhibitory activity suggesting that epitopes on the conserved face can be targets of neutralizing antibodies, despite being under less immune pressure than immunodominant polymorphic regions.

For *Pv*DBP, one of the regions of high diversity across all populations was a previously identified epitope within subdomain 2 termed the DEK epitope [33]. Others have observed that the DEK epitope is highly polymorphic and immunodominant [44]. However, due to the polymorphic nature of this epitope, cross-strain specificity is a concern when creating a *Pv*DBP RII-based vaccine. Recent work has characterized the location of several conserved epitopes within *Pv*DBP RII that are the target of inhibitory mAbs 2D10, 2H2 and 2C6 [73, 74], and all of these conserved epitopes fall within subdomain III, which had the lowest overall nucleotide diversity in this current study. This highlights some of the limitations of using population-level genomic data for identification of functionally important targets of antibody responses—the possibility that conserved regions may contain potential epitopes that are the targets of inhibitory antibodies cannot be excluded.

Several attempts have been made to divert immune responses away from these highly polymorphic regions of *Pv*DBP RII and towards conserved epitopes. One attempt to focus away from polymorphic regions involved mutating residues in the DEK epitope to reduce its immunogenicity, and these DEKnull mutants induce antibodies that bind *Pv*DBP and can inhibit the interaction with Duffy Binding Ligand [33]. More recently, further epitope focusing techniques have been employed with *Pv*DBP RII, with one strategy involving mutation of all polymorphic residues to alanine, threonine or serine residues [75]. This ‘DEKnull-2’ recombinant *Pv*DBP RII construct was shown to elicit broadly neutralizing antibodies following mouse immunization, with some naturally exposed individuals also shown to recognize the conserved epitopes on this construct [75]. Other recent efforts towards the development of a *Pv*DBP RII vaccine include a Phase 1a trial of a prime-boost viral-vectored vaccine that demonstrated both safety and immunogenicity, with cross-strain inhibition demonstrated for the single heterologous strain tested [76].

Given the success using epitope focusing techniques for *Pv*DBP RII, we suggest that such an approach could be applied to AMA1 to focus antibody responses towards conserved epitopes on the silent face of DI or within DIII. This would involve mutating major polymorphic residues to reduce the immunodominance of epitopes within the polymorphic face of DI; the most polymorphic residues identified in this study (Additional file 11) could serve as starting point for this work. Alternatively, it has been shown for *Pf*AMA1 that immunization with multiple heterologous strains of *Pf*AMA1 is capable of inducing strain transcending antibody responses [24], and this approach could also be applied to *Pv*AMA1. Other epitope-focusing approaches also exist, including the use of small protein scaffolds to mimic native epitopes [66, 67], although these might be challenging given the discontinuous nature of many potential epitopes within AMA1. The approaches used in this work could also be applied to other antigens such as *Pv*RBP2b, which has recently been identified as a ligand for reticulocyte invasion via binding to transferrin receptor 1 (TfR1) [77].

## Conclusions

In this work, signatures of diversity and selection were identified on *Pv*AMA1 and *Pv*DBP, and it was shown that the regions of high diversity and balancing selection on the protein structure are remarkably similar across a number of populations. This suggests that dominant epitopes are the same across multiple human populations, which has positive implications for the development of a universal *P. vivax* vaccine. Furthermore, polymorphisms were observed to cluster around binding interfaces on both *Pv*DBP and *Pv*AMA1, suggesting a level of immune pressure on residues surrounding these key binding interfaces. Large regions with very low diversity were also identified for both antigens, and it is suggested that these areas may also be useful targets to focus on for further vaccine development, given previous evidence of functional antibody responses against these conserved regions.

## Additional files

**Additional file 1.** Domains/subdomains of *Pv*AMA1.

**Additional file 2.** Domains/subdomains of *Pv*DBP RII.

**Additional file 3.** Spatially-derived nucleotide diversity for *Pv*AMA1 across multiple populations.

**Additional file 4.** Spatially-derived Tajima’s D for *Pv*AMA1 across multiple populations.

**Additional file 5.** Spatially-derived nucleotide diversity for *Pv*DBP across multiple populations.

**Additional file 6.** Spatially-derived Tajima’s D for *Pv*DBP across multiple populations.

**Additional file 7.** Location of statistically significant (p < 0.05) Tajima’s D values on modelled *Pv*AMA1 (a) and *Pv*DBP (b) structures.

**Additional file 8.** Sequence diversity at interfaces.

**Additional file 9.** Comparison of spatially derived Tajima’s D and conventional linear sliding window calculation of Tajima’s D for *Pv*AMA1.

**Additional file 10.** Comparison of spatially derived Tajima’s D and conventional linear sliding window calculation of Tajima’s D for *Pv*DBP RII.

**Additional file 11.** Table of highly polymorphic residues within *Pv*AMA1.
