# Supplementary material for: Structural patterns of selection and diversity for Plasmodium vivax antigens DBP and AMA1
Source: Malar J. 2018 May 2;17:183. doi: 10.1186/s12936-018-2324-3 (PMC5930944; doi:10.1186/s12936-018-2324-3)
Supplement: Supplementary file 1 — Additional file 1. Domains/subdomains of PvAMA1. [file 12936_2018_2324_MOESM1_ESM.pdf]

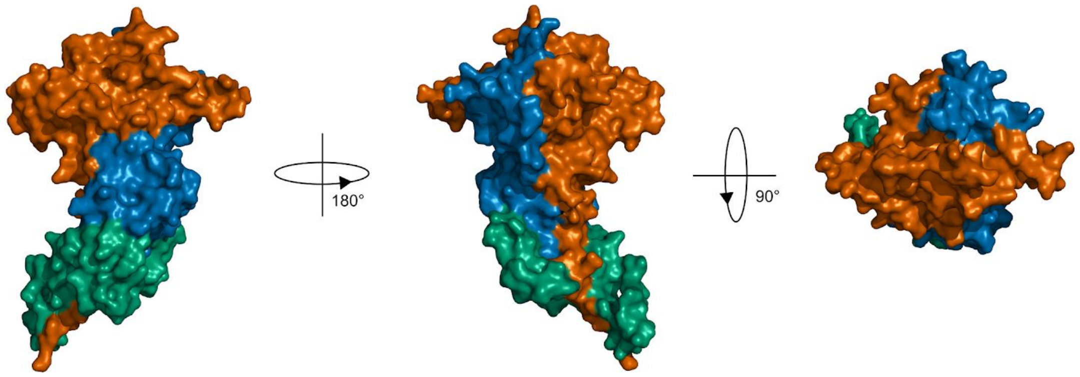

Additional File 1: Domains/subdomains of *Pv*AMA1. *Pv*AMA1 has been divided into three domains, termed Domain I (DI), II (DII) and III (DIII), shown in orange, blue and green, respectively. Domain assignment follows that outlined by Pizarro *et al.* [21], and corresponds to the following residues in the Sal-1 reference sequence: 41-250 (DI); 251-387 (DII); 388-474 (DIII).
