# Supplementary material for: Structural patterns of selection and diversity for Plasmodium vivax antigens DBP and AMA1
Source: Malar J. 2018 May 2;17:183. doi: 10.1186/s12936-018-2324-3 (PMC5930944; doi:10.1186/s12936-018-2324-3)
Supplement: Supplementary file 2 — Additional file 2. Domains/subdomains of PvDBP RII. [file 12936_2018_2324_MOESM2_ESM.pdf]

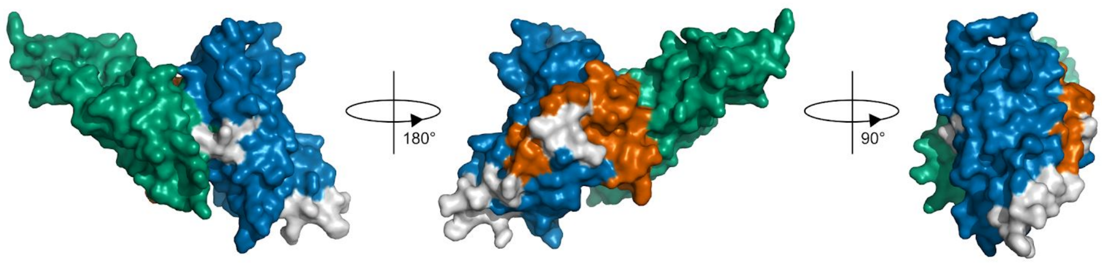

Additional File 2: Domains/subdomains of *Pv*DBP RII. *Pv*DBP RII has been divided into three subdomains, indicated in orange (subdomain 1), blue (subdomain 2) and green (subdomain 3). Subdomain assignment follows that outlined by Singh *et al.* [41], corresponding to the following residues in the Sal-1 reference sequence: 216-253 (subdomain 1); 265-381 (subdomain 2); 387-508 (subdomain 3). Linkers between subdomains are shown in white.
