# Supplementary material for: Structural patterns of selection and diversity for Plasmodium vivax antigens DBP and AMA1
Source: Malar J. 2018 May 2;17:183. doi: 10.1186/s12936-018-2324-3 (PMC5930944; doi:10.1186/s12936-018-2324-3)
Supplement: Supplementary file 3 — Additional file 3. Spatially-derived nucleotide diversity for PvAMA1 across multiple populations. [file 12936_2018_2324_MOESM3_ESM.pdf]

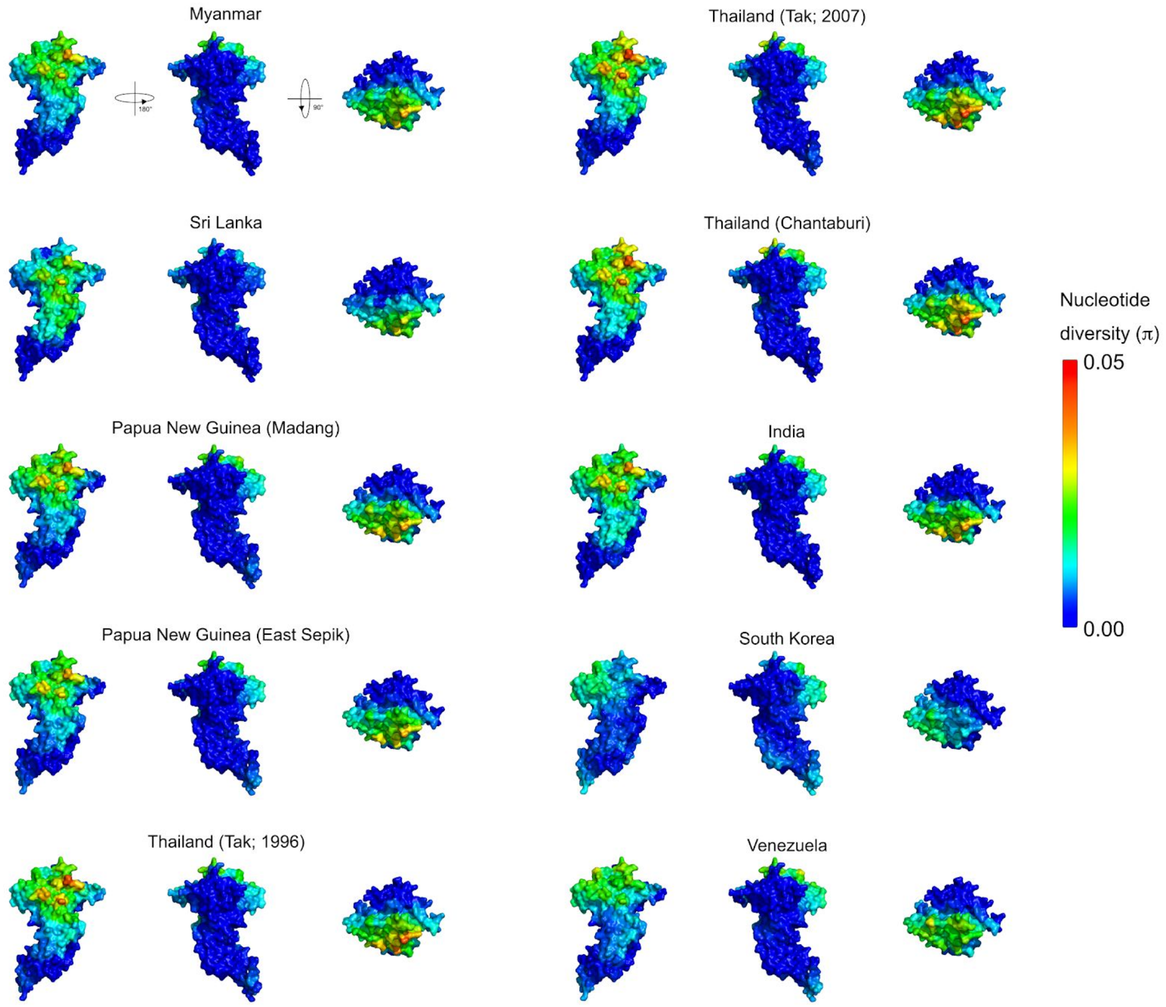

Additional File 3: Spatially-derived nucleotide diversity for *Pv*AMA1 across multiple populations. Nucleotide diversity was calculated using a 3D sliding window over a modelled *Pv*AMA1 structure, with a radius of 15 Å for each window.
