# Supplementary material for: Structural patterns of selection and diversity for Plasmodium vivax antigens DBP and AMA1
Source: Malar J. 2018 May 2;17:183. doi: 10.1186/s12936-018-2324-3 (PMC5930944; doi:10.1186/s12936-018-2324-3)
Supplement: Supplementary file 4 — Additional file 4. Spatially-derived Tajima’s D for PvAMA1 across multiple populations. [file 12936_2018_2324_MOESM4_ESM.pdf]

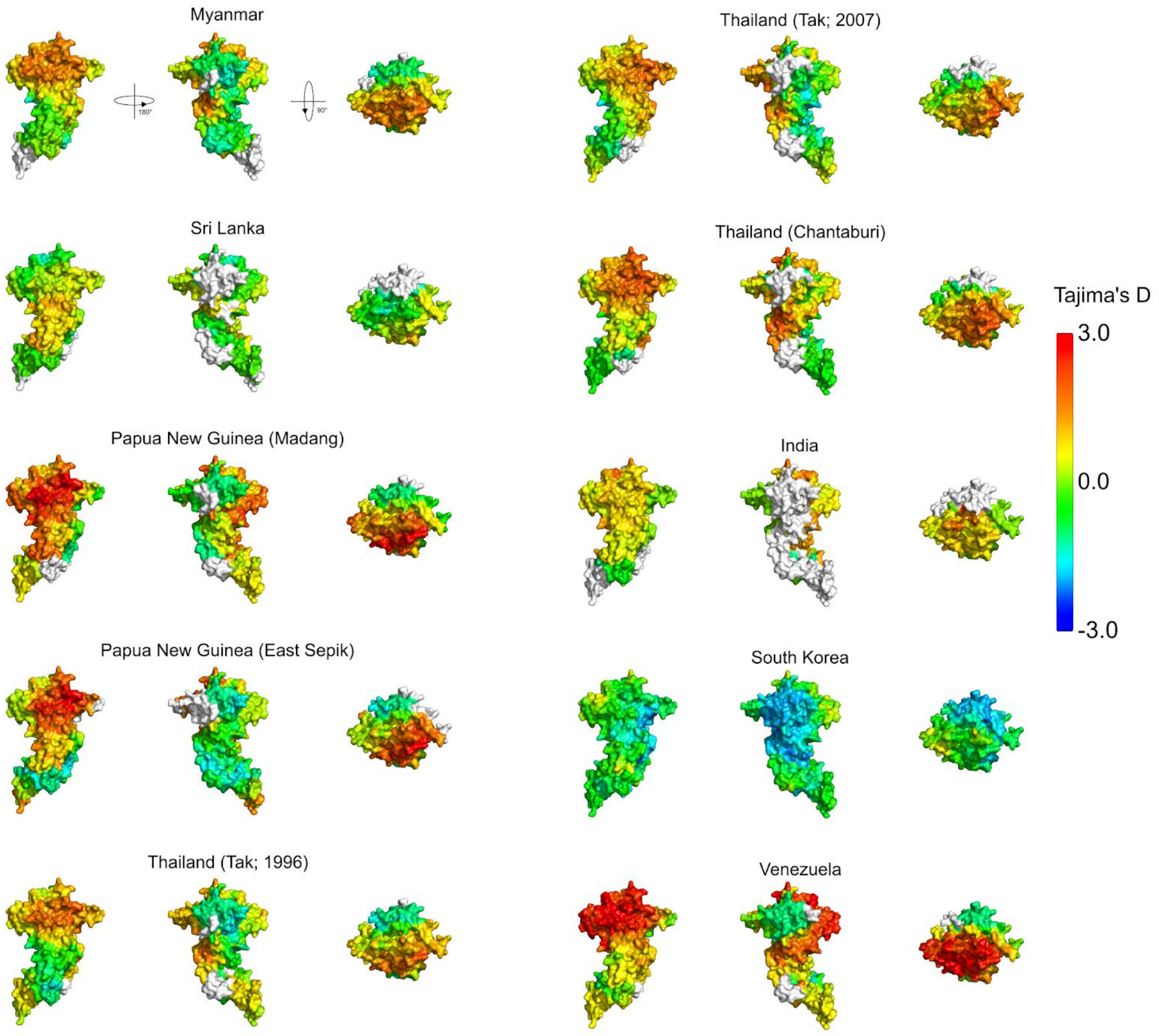

Additional File 4: Spatially-derived Tajima's D for *Pv*AMA1 across multiple populations. Tajima's D was calculated using a 3D sliding window over a modelled *Pv*AMA1 structure, with a radius of 15 Å for each window.
