# Supplementary material for: Structural patterns of selection and diversity for Plasmodium vivax antigens DBP and AMA1
Source: Malar J. 2018 May 2;17:183. doi: 10.1186/s12936-018-2324-3 (PMC5930944; doi:10.1186/s12936-018-2324-3)
Supplement: Supplementary file 5 — Additional file 5. Spatially-derived nucleotide diversity for PvDBP across multiple populations. [file 12936_2018_2324_MOESM5_ESM.pdf]

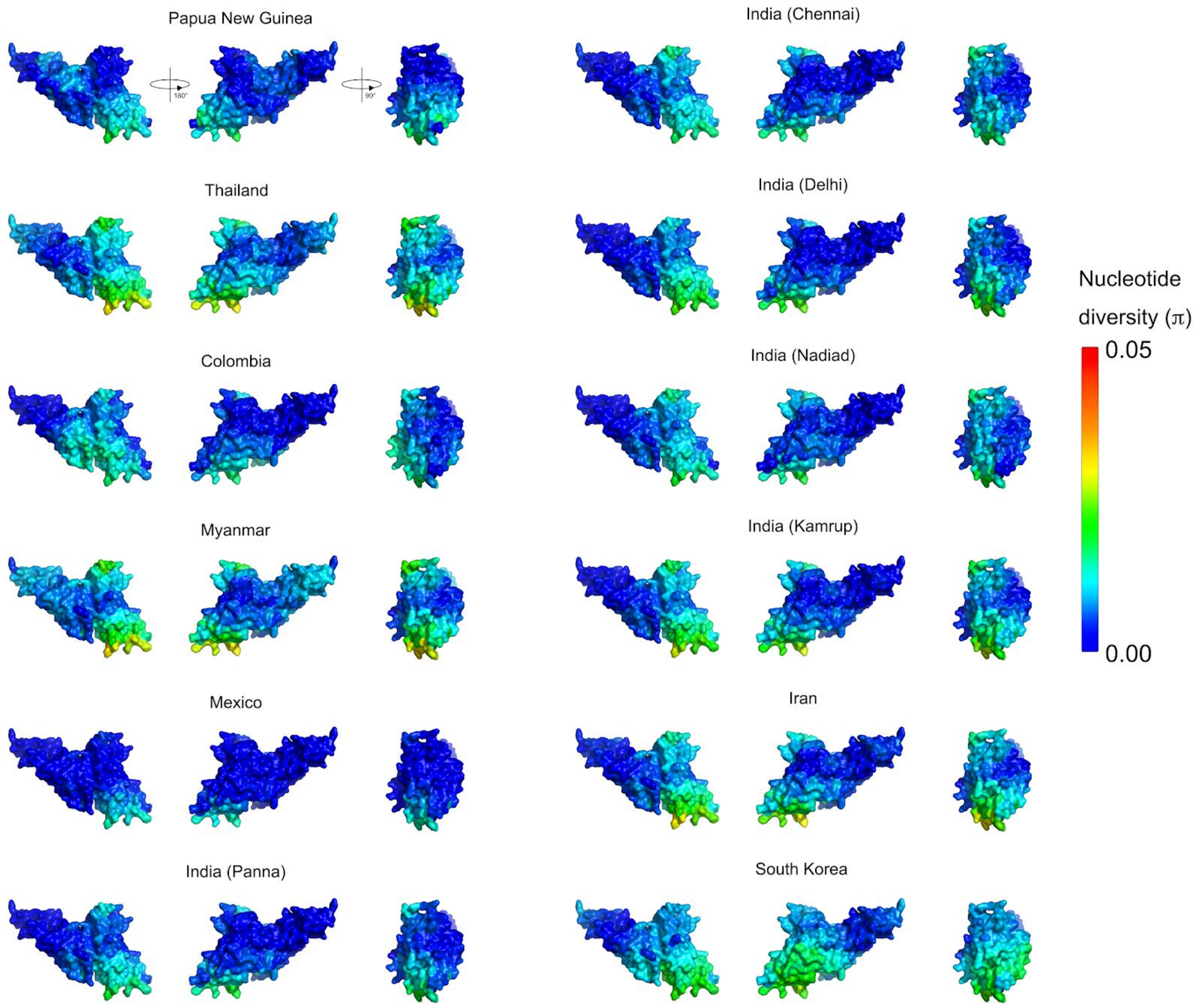

Additional File 5: Spatially-derived nucleotide diversity for *Pv*DBP across multiple populations. Nucleotide diversity was calculated using a 3D sliding window over a modelled *Pv*DBP structure, with a radius of 15 Å for each window.
