# Supplementary material for: Structural patterns of selection and diversity for Plasmodium vivax antigens DBP and AMA1
Source: Malar J. 2018 May 2;17:183. doi: 10.1186/s12936-018-2324-3 (PMC5930944; doi:10.1186/s12936-018-2324-3)
Supplement: Supplementary file 6 — Additional file 6. Spatially-derived Tajima’s D for PvDBP across multiple populations. [file 12936_2018_2324_MOESM6_ESM.pdf]

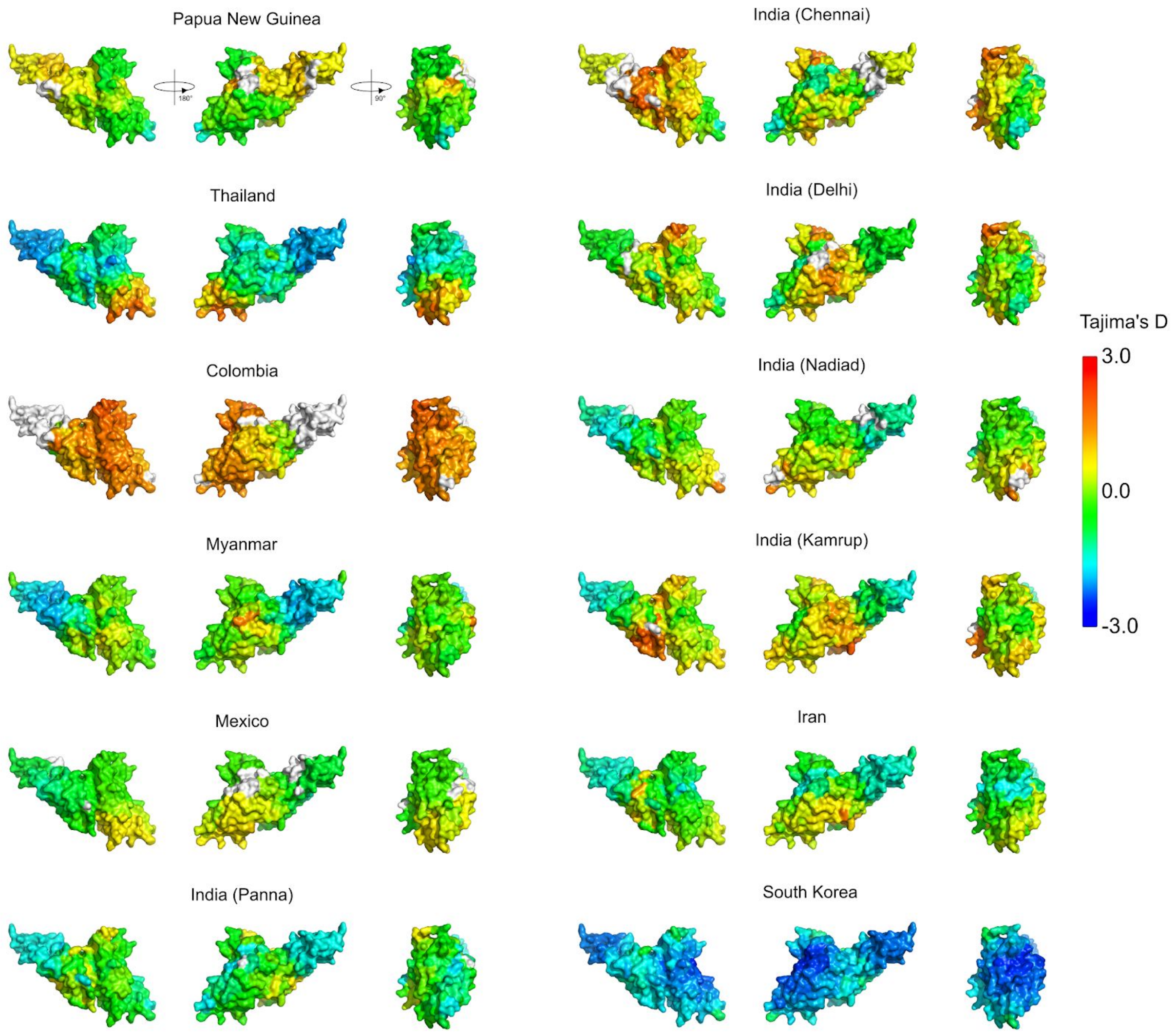

Additional File 6: Spatially-derived Tajima's D for *Pv*DBP across multiple populations. Tajima's D was calculated using a 3D sliding window over a modelled *Pv*DBP structure, with a radius of 15 Å for each window.
