# Supplementary material for: Structural patterns of selection and diversity for Plasmodium vivax antigens DBP and AMA1
Source: Malar J. 2018 May 2;17:183. doi: 10.1186/s12936-018-2324-3 (PMC5930944; doi:10.1186/s12936-018-2324-3)
Supplement: Supplementary file 7 — Additional file 7. Location of statistically significant (p < 0.05) Tajima’s D values on modelled PvAMA1 (a) and PvDBP (b) structures. [file 12936_2018_2324_MOESM7_ESM.pdf]

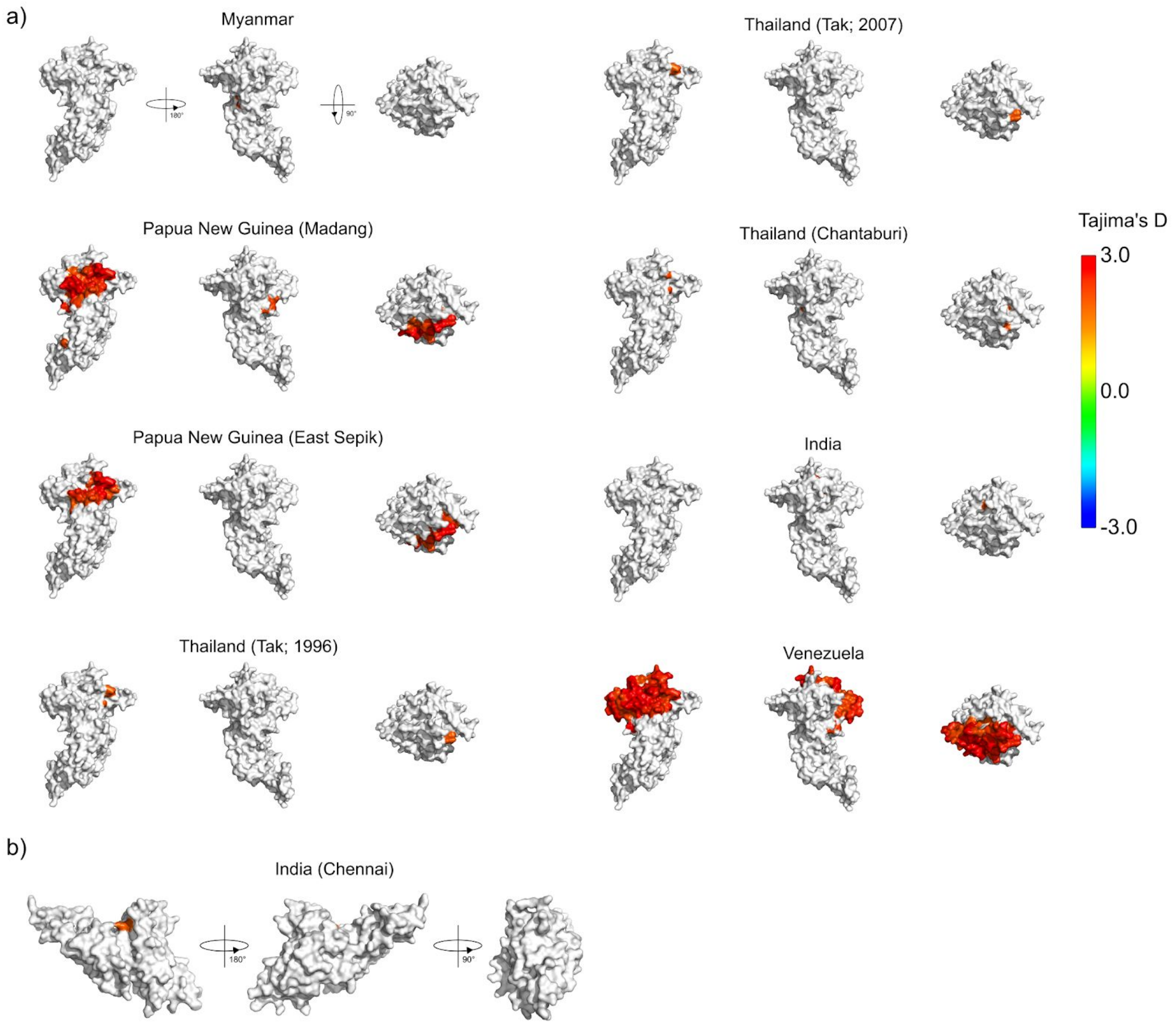

Additional File 7: Location of statistically significant ($p < 0.05$) Tajima's D values on modelled *Pv*AMA1 (a) and *Pv*DBP (b) structures. Confidence limits are those defined by Tajima [60]. Only populations with significant Tajima's D values are shown here.
