# Supplementary material for: Structural patterns of selection and diversity for Plasmodium vivax antigens DBP and AMA1
Source: Malar J. 2018 May 2;17:183. doi: 10.1186/s12936-018-2324-3 (PMC5930944; doi:10.1186/s12936-018-2324-3)
Supplement: Supplementary file 8 — Additional file 8. Sequence diversity at interfaces. [file 12936_2018_2324_MOESM8_ESM.pdf]

Additional File 8: Sequence diversity at interfaces.

***Pv*DBP**		
**Polymorphism**	**Minor Allele Count**	**Minor Allele Frequency (%)**
**R263S**	**49**	**20.1**
T359R	10	4.1
V240L	3	1.2
V240I	1	0.4
I265L	1	0.4
K366N	1	0.4
Y271S	1	0.4
***Pv*AMA1**		
**Polymorphism**	**Minor Allele Count**	**Minor Allele Frequency (%)**
**N132D**	**235**	**46.4**
**N130K**	**57**	**11.3**
D133N	17	3.4
M153T	12	2.4
G117R	12	2.4
K86R	3	0.6
A172T	3	0.6
N132G	2	0.4
R88G	1	0.2

Note: The presence of polymorphisms was determined using sequences from all populations. Polymorphisms with a minor allele frequency > 5% are shown in bold. Any polymorphic residues within 4 Å of the *Pf*AMA1:RON2 interface or the *Pv*DBP dimerization/DARC binding interface are included in the above table.
