# Supplementary material for: Structural patterns of selection and diversity for Plasmodium vivax antigens DBP and AMA1
Source: Malar J. 2018 May 2;17:183. doi: 10.1186/s12936-018-2324-3 (PMC5930944; doi:10.1186/s12936-018-2324-3)
Supplement: Supplementary file 10 — Additional file 10. Comparison of spatially derived Tajima’s D and conventional linear sliding window calculation of Tajima’s D for PvDBP RII. [file 12936_2018_2324_MOESM10_ESM.pdf]

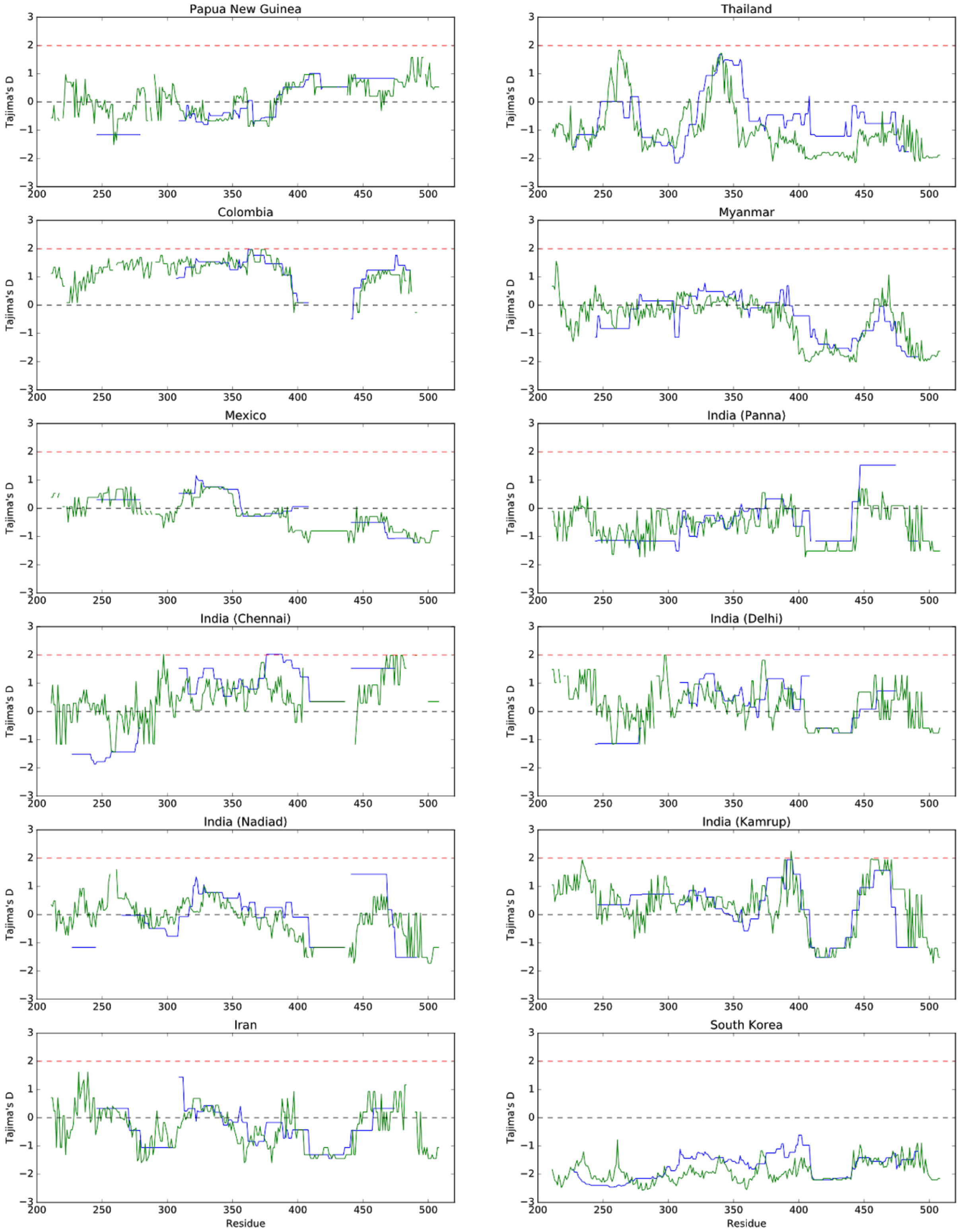


**Additional File 10: Comparison of spatially derived Tajima's D and conventional linear sliding window calculation of Tajima's D for *Pv*DBP RII.** Tajima's D values using a linear sliding window results are shown in blue, whilst spatially derived Tajima's D values are shown in green. The threshold for significance ($p < 0.05$) as defined by Tajima [60] is shown as a dotted red line.
